# Production of glucosinolates, phenolic compounds and associated gene expression profiles of hairy root cultures in turnip (*Brassica rapa* ssp. *rapa*)

**DOI:** 10.1007/s13205-016-0492-9

**Published:** 2016-08-20

**Authors:** Ill-Min Chung, Kaliyaperumal Rekha, Govindasamy Rajakumar, Muthu Thiruvengadam

**Affiliations:** Department of Applied Bioscience, College of Life and Environmental Sciences, Konkuk University, Seoul, 143 701 Republic of Korea

**Keywords:** Anticancer activity, Antimicrobial activity, Biosynthetic gene expression, Glucosinolates, Hairy roots, Phenolic compounds

## Abstract

Turnip (*Brassica rapa* ssp. *rapa*) is an important vegetable crop producing glucosinolates (GSLs) and phenolic compounds. The GSLs, phenolic compound contents and transcript levels in hairy root cultures, as well as their antioxidant, antimicrobial and anticancer activity were studied in turnip. Transgenic hairy root lines were confirmed by polymerase chain reaction (PCR) and reverse transcription-PCR. GSLs levels (glucoallysin, glucobrassicanapin, gluconasturtiin, glucobrassicin, 4-methoxyglucobrassicin, neoglucobrassicin and 4-hydroxyglucobrassicin) and their gene expression levels (*BrMYB28*, *BrMYB29*, *BrMYB34*, *BrMYB51, BrMYB122*, *CYP79* and *CYP83*) significantly increased in hairy roots compared with that in non-transformed roots. Furthermore, hairy roots efficiently produced several important individual phenolic compounds (flavonols, hydroxybenzoic and hydroxycinnamic acids). Colorimetric analysis revealed that the highest levels of total phenol, flavonoid contents, and their gene expression levels (*PAL*, *CHI* and *FLS*) in hairy roots than non-transformed roots. Our study provides beneficial information on the molecular and physiological active processes that are associated with the phytochemical content and biosynthetic gene expression in turnip. Moreover, antioxidant activity, as measured by DPPH scavenging activity, reducing potential, phosphomolybdenum and ferrous ion chelating ability assays was significantly higher in hairy roots. Hairy root extracts exhibited higher antimicrobial activity against bacterial and fungal species. The extract of hairy roots showed inhibition of human breast and colon cancer cell lines.

## Introduction

Cruciferous vegetables are a major food source all over the world, and they contain important phytochemicals such as glucosinolates (GSLs) and their breakdown products. GSLs are nitrogen (N)- and sulfur (S)-rich anionic phytochemicals that are derived from a glucose molecule and an amino acid, and are also known as β-thioglucoside-N-hydroxysulfates (Bennett et al. 2004). GSLs have been classified into three major groups, namely, aliphatic (AGSLs), aromatic, and indolic glucosinolates (IGSLs), derived from the amino acids of methionine, phenylalanine, and tryptophan, respectively (Kastell et al. 2015). Epidemiological studies demonstrate that a high consumption of cruciferous vegetables significantly reduces the risk of certain cancers and cardiovascular diseases, presumably because GSLs breakdown products and act as chemoprotective agents (Hong and Kim 2008; Thiruvengadam and Chung 2015). Turnip (*Brassica rapa* ssp. *rapa*), which belongs to the Cruciferae family, is one of the most important leaf and root vegetable crops for human consumption, and it is also used for animal fodder. Turnip roots contain high amounts of GSLs, phenolic compounds and other bioactive compounds (Zhang et al. 2008; Thiruvengadam and Chung 2015). GSLs biosynthesis is positively regulated by various R2R3 MYB transcription factors. *MYB34*, *MYB51*, and *MYB122* are involved in the regulation of IGSLs biosynthesis, while *MYB28*, *MYB29*, and *MYB76* are participating in the regulation of AGSLs biosynthesis in *Arabidopsis* (Sønderby et al. 2010). The core structures are formed via oxidation by cytochrome P450 enzymes (CYP79 and CYP83), followed by C-S cleavage, glucosylation, and sulfation. *CYP79F1* oxidizes all methionine derivatives with a preference for short-chain precursors and is involved in the biosynthesis of AGSLs. *CYP83B1* has a high affinity for the indole-3-acetaldoxime derived from tryptophan and for aromatic aldoximes derived from phenylalanine or tyrosine and involved in the biosynthesis of IGSLs (Kastell et al. 2015). Phenylpropanoids are a large group of phytochemicals formed by plants and are derived from the amino acid precursors, and have been reported to have multiple biological effects, including antioxidant activity (Thiruvengadam and Chung 2015). Flavonoids are synthesized through the phenylpropanoid pathway, converting phenylalanine into 4-coumaroyl-CoA, which finally enters the flavonoid biosynthesis pathway.

The production of bioactive compounds through field grown of intact plants has various disadvantages, including low yields, slow growth cycles, and fluctuations in quantity due to unfavorable environmental conditions, infestation, and disease. Hairy root culture technology is an attractive alternative system for the uniform production of bioactive compounds, can continuously provide high-value medicines, foods, and healthy constituents, independent of geographical, climatic, or environmental variations and constraints (Wilson and Roberts 2014). Hairy root cultures produced via *Agrobacterium rhizogenes*-mediated transformations have emerged as an ideal biotechnological system for the production of valuable phytochemicals because of their genetic stability and large-scale biomass production without phytohormones (Chandra and Chandra 2011). The greatest benefit of hairy roots is that they frequently exhibit about the same or superior biosynthetic capacity for secondary metabolite production as their parent plants (Kim et al. 2002; Chandra and Chandra 2011). Hairy root cultures have been commonly studied for the production of bioactive compounds useful as medicines, cosmetics, and food additives (Georgiev et al. 2007; Medina-Bolivar et al. 2007; Majumdar et al. 2011; Nagella et al. 2013; Thiruvengadam et al. 2014). Root loci (*rol*) genes harbored by the root-inducing (Ri) plasmid of *A. rhizogenes* are integrated into the host plant genome, causing hairy root (Chandra 2012). The *rol* genes are thought to affect the growth and development of transformed roots and to induce the secondary metabolite synthesis by activating transcription defense genes. Previously it was reported that *rolC* from *A. rhizogenes* T-DNA could stimulate the production of secondary metabolites in the transformed cells of different plants (Shkryl et al. 2008). Earlier limited studies have been reported in hairy root induction of turnip (Tanaka et al. 1985; Huet et al. 2014). However, only a few studies have been described in the individual GSLs content of hairy root cultures in *Nasturtium officinale* (Park et al. 2011), *Sinapis alba* and *B. rapa* (Kastell et al. 2013b), *B. oleracea* var. *italica* (Kim et al. 2013), and *Arabidopsis thaliana* (Kastell et al. 2015). In this study, we successfully established, for the first time, hairy root cultures to assess the production of GSLs (aliphatic, aromatic and indolic) and phenolic compound (flavonols, hydroxycinnamic and hydroxybenzoic acids) content in turnip. In addition, we studied the GSLs and phenolic compound gene expression levels of hairy roots and non-transformed roots in turnip. Finally, we evaluated the total phenolic, flavonoid content, and biological (antioxidant, antimicrobial and anticancer) activities of hairy roots and non-transformed roots of turnip.

## Materials and methods

### Plant material

Seeds of *B. rapa* ssp*. rapa* were received from Kyoung Shin Seeds Co., Ltd., Korea. Seeds were surface-sterilized with 70 % (v/v) ethanol for 1 minute, and washed twice with sterile distilled water. The seeds were then soaked in 2 % sodium hypochlorite and stirred for 10 minutes followed by brief washing in sterile deionized water. Disinfected seeds were germinated on MS medium (Murashige and Skoog 1962) with 0.8 % agar and 3.0 % sucrose. The cultures were kept under a 16 hour photoperiod (30 μmol m^−2^ s^−1^) provided by cool white fluorescent lamps at 25 ± 1 °C. Explants from roots, hypocotyl, and leaves of 7-day-old seedlings were used for transformation.

### Establishment of hairy root cultures

The leaves, hypocotyls, and roots were directly wounded with sterile needles containing overnight bacterial suspension (OD600 nm = 1.0) of the *Agrobacterium rhizogenes* strain KCTC 2703. Explants were given bacterial infection for 30 min; then explants were dried on sterilized filter paper for 3 min and then placed on MS solid medium and incubated in the dark at 25 ± 1 °C. After 3 days of co-cultivation, the explants were rinsed with sterile distilled water containing 300 mg L^−1^ cefotaxime to eliminate the residual bacteria from the explants. The explants were transferred to MS solid medium supplemented with 300 mg L^−1^ cefotaxime. Root cultures were incubated under 16 hour light/8 hour dark provided by cool white fluorescent tubes (30 μmol m^−2^ s^−1^) at 25 ± 1 °C. After 21 days, 300 mg fresh mass (FM) of roots were subcultured individually into MS liquid medium, supplemented with 4 % sucrose and 300 mg L^−1^ cefotaxime. Cultures were kept on an orbital shaker (100 rpm) and incubated under the same conditions. Roots were subcultured every 12 days. Cefotaxime concentration was regularly declined and finally removed from the culture medium. Non-transformed roots were excised from in vitro germinated seedlings grown in MS liquid medium devoid of plant growth regulators.

### Growth kinetics and nutrient consumption in the hairy root cultures

Hairy roots (300 mg) were cultured in MS liquid medium supplemented with 4 % sucrose. Growth kinetics at various time intervals (6, 12, 18, 25, and 30 days) was investigated to optimize biomass accumulation. Full-strength and half-strength Murashige and Skoog (1962), Nitsch and Nitsch (1969), Gamborg et al. (1968), and Linsmaier and Skoog (1965) media, as well as different concentrations of sucrose (1, 2, 3, 4, and 5 %), were tested to find a combination that resulted in the highest root biomass accumulation. The cultures were placed under continuous agitation at 100 rpm on an orbital shaker and incubated under the above mentioned culture conditions. After 25 days of culture, the biomass of the hairy roots was evaluated. The roots were separated from the media and their fresh mass (FM) was determined after they were washed with distilled water and the excess surface water blotted away. The dry mass (DM) was determined after lyophilization.

### Molecular analysis of hairy roots

#### DNA extraction and polymerase chain reaction (PCR)

Total genomic DNA was isolated from non-transgenic and transgenic hairy root lines using the cetyltrimethylammonium bromide (CTAB) method (Khan et al. 2007). Isolated genomic DNA was used in PCR analysis for detecting the *rolC* gene. PCR analysis was performed using a programmed DNA thermal cycler (PerkinElmer, USA). The *rolC* and *virD2* genes using specific primers according to previous reports by Sivakumar et al. (2005) and Medina-Bolivar et al. (2007). PCR was carried out in the entire volume of 25 µL reaction mixtures containing 100 ng total DNA of samples, 200 µM dNTPs, 2 µL 10 × PCR buffer, 0.8 µL MgCl_2_ 1.25 units of Taq DNA polymerase and 25 pmol of each primer. In the PCR program, DNA was denatured at 94 °C for 4 min, followed with 30 amplification cycles (94 °C for 1 min, 60 °C for 1 min, 72 °C for 1 min), and with finishing extension at 72 °C for 5 min. For *virD2* the conditions were: initial denaturation at 95 °C for 3 min, followed by 30 amplification cycles (95 °C for 30 s, 56 °C for 30 s, and 72 °C for 45 s), and 10 min at 72 °C. The referred primer sequences of *rolC* were used for confirmation of transformed hairy roots and the *virD2* gene was taken as evidence against contamination by *Agrobacterium*.

#### Reverse transcription PCR (RT-PCR)

Total RNA was isolated from non-transgenic and transgenic hairy root lines using the RNeasy mini kit. RT-PCR analysis was carried out with a Revert-Aid™ first strand complementary DNA (cDNA) synthesis kit (Fermentas Life Sciences, USA) following the manufacturer’s instructions. The same PCR primers for the *rolC* gene were used with similar conditions for RT-PCR (Thiruvengadam et al. 2014).

### Analytical methods of bioactive compounds

#### Extraction of glucosinolates (GSLs)

The lyophilized powder of transgenic and non-transgenic roots was extracted as described earlier by Thiruvengadam and Chung (2015). One-hundred milligrams of lyophilized root powder samples were extracted with EtOH (70 % v/v, 1.5 mL) in a boiling water bath (70 °C) followed by centrifugation at 13,000*g* and 4 °C for 20 min. This process was repeated for three times to allow the complete extraction of the residue and the supernatants obtained from each extraction were combined. The combined extract was loaded onto a mini-column filled with DEAE Sephadex A-25, 500 μL of aryl sulfatase was injected onto the column for overnight desulfation at room temperature. Desulfo-glucosinolates (DS-GSLs) were then eluted into a micro-centrifuge tube (2.0 mL) using three aliquots (0.5 mL each) of distilled water and filtered through a 0.2-μm PTFE syringe filter.

#### Estimation of desulfo-glucosinolates (DS-GSLs) using ultra-high-pressure liquid chromatography–triple quadrupole mass spectrometry (UHPLC-TQMS)

DS-GSLs was analyzed using an EVOQ advanced UHPLC system (CTC PAL-xt Autosampler; Bruker, USA). Samples were separated on a C_18_ column (50 mm × 2 mm × 3 μm; YMC, USA) with water (mobile A) and acetonitrile (mobile B) containing 0.1 % formic acid. Five microliters of the sample was injected, and the flow rate was maintained at 0.2 mL min^−1^. Heated electrospray ionization (HESI) was performed in the negative (−) and positive (+) ion mode within a range of 0–5000 *m*/*z*. The operating parameters were: ion source temperature, 400 °C; cone gas flow, 60 L h^−1^; desolvation gas flow, 600 L h^−1^; capillary voltage, 5.0 kV; and cone voltage, up to 35 V.

#### Extraction and estimation of total phenolic contents (TPC)

Total phenolic content was estimated by the Folin–Ciocalteu colorimetric method with slight modifications of Thiruvengadam et al. (2014). Briefly, 100 µL of the transgenic and non-transgenic root extracts were mixed with 3.10 mL of distilled water, followed by addition of 0.2 mL Folin–Ciocalteu reagent. After 5 min, 0.6 mL of 20 % sodium carbonate solution was added. The absorbance of the resulting blue-coloured solution was measured at 760 nm using a UV–visible spectrophotometer (UV-2120 Optizen, Mecasys, Korea) after incubation at 30 °C for 1 h with intermittent shaking. The concentration of the TPC was calculated as mg of gallic acid equivalent by using an equation obtained from the gallic acid calibration curve.

#### Extraction and estimation of total flavonoid contents (TFC)

The total flavonoid content (TFC) of the transgenic and non-transgenic root extracts using the aluminum chloride colorimetry method described by Thiruvengadam et al. (2014) with slight modifications. In brief, the extract (0.2 mL) with 0.1 mL of 10 % (w/v) aluminum chloride solution, 0.1 mL of 1 M potassium acetate solution, and distilled water (4.6 mL) were mixed. After incubation at room temperature for 30 min, the absorbance was measured at 415 nm using a UV–visible spectrophotometer (UV-2120 Optizen). Quercetin was used to construct the calibration curve.

#### Extraction and estimation of phenolic compounds using ultra-performance liquid chromatography (UPLC)

Lyophilized powder (1 g) of the transgenic and non-transgenic roots was extracted according to the previously described method (Thiruvengadam et al. 2014). The filtrate was analyzed with a Thermo Accela UPLC system (Thermo, USA). The separation was achieved using a HALO C_18_ (2.7 µm, 2.1 × 100 mm) column and the absorbance was measured at 280 nm. The mobile phases were 0.1 % glacial acetic acid in distilled water (solvent A) and 0.1 % glacial acetic acid in acetonitrile (solvent B). The gradient procedure was described earlier (Thiruvengadam et al. 2014). The pure standard solutions (25, 50, 100, and 150 µg mL^−1^) of protocatechuic acid, gentisic acid, syringic acid, *p*-hydroxybenzoic acid, formononetin, *p*-coumaric acid, *m*-coumaric acid, chlorogenic acid, ferulic acid, rutin, *t*-cinnamic acid, myricetin, catechin, quercetin, naringenin, kaempferol, biochanin A, and hesperidin (Sigma-Aldrich, St. Louis, MO, USA) were dissolved in methanol and analyzed the samples. Phenolic compounds of hairy roots and non-transformed root extracts were identified based on the retention time and UV spectra of the standards, while the quantitative data were calculated based on the calibration curves of the individual standards. Results were expressed as µg g^−1^ of each compound in the total phenolic content.

### Transcript quantification using real-time polymerase chain reaction (qRT-PCR) assays

Changes in transcript levels of genes related to GSLs (*BrMYB28*, *BrMYB29*, *BrMYB34*, *BrMYB51*, *BrMYB122*, *CYP79*, and *CYP83*) and phenolic (*PAL*, *CHI* and *FLS*) biosynthesis were quantified by qPCR. Total RNA was isolated from hairy roots and non-transformed roots using a NucleoSpin RNA Plant mini prep kit (Macherey-Nagel, Germany) according to the manufacturer’s protocol. The integrity of the extracted RNA was determined through RNA denaturing gel electrophoresis and then the purity as well as the concentration of RNA were measured by a spectrophotometer (Nanodrop ND-1000 Spectrophotometer; Celbio, Italy). The cDNA synthesis was carried out using 3 µg total RNA, 25 pM oligodT primer, and RT-premix (Bioneer, Korea) according to the manufacturer’s instructions. qPCR was performed in the CFX 96 Real-Time PCR system (BioRad, USA) using the AccuPower 2X GreenStar qPCR kit (Bioneer, Korea). The qPCR cycling conditions were 95 °C for 5 min, followed by 35 cycles of 94 °C for 30 s, annealing temperature for 30 s, and an extension at 72 °C for 2 min. The primers used in the qPCR analysis are given in Table 1. Primer specificity and amplification efficiency were determined by melting curve analysis. Actin gene was used as the internal control for normalization. Normalized fold change in expression was calculated by using the 2^−ΔΔCt^ method (Thiruvengadam and Chung 2015).

**Table 1:** Primers for associated genes involved in glucosinolates and phenolic biosynthetic pathway

No.	Database	Gene accession number	Gene name	Primer sequence (5′–3′)
1	NCBI	FJ584287	*BrMYB28*	F: AAGAAAGCCATGTTGTGTCG
				R: TTTCCACACCTTTTCAACCC
2	NCBI	FJ584290	*BrMYB29*	F: AGTTGTAGATTGCGATGGGC
				R: CGTTGTCTGTCCTTTTGGGC
3	NCBI	FJ584296	*BrMYB51*	F: AGATGTGGCAAAAGC TGCAGA
				R: GGTAATCCACGAGCT ATTGCA
4	NCBI	FJ584293	*BrMYB34*	F: ACTCTCCCGGAAAAA GCTGGAT
				R: CGTTATCAGTTCGTCCAGCCA
5	NCBI	FJ584300	*BrMYB122*	F: AACCACTGGAACACTCACATCAA
				R: TACCAAATCTGTTTGCTACTCTGTTCA
6	NCBI	FJ376051	*CYP83B1*	F: TCCTTCGGAGCTCCACGAAGAAAT
				R: ACTCAGCCGATGAGATCACTGCAA
7	BRAD	Bra026058	*CYP79F1*	F: CTGCAGGAGTTTTGTATAGCAGC
				R: CTGCAACAAGCTTTTAAGTAGTTTAGA
8	NCBI	AY055752	*PAL*	F: AGAACGGTGTCGCTCTTCAG
				R: TGTGGCGGAGTGTGGTAATG
9	BRAD	Bra034304	*CHI*	F: TGGTGGCCTAGACAACGATGAGTT
				R: TCACACTCCCAACTTGGTTTCCCT
10	BRAD	Bra038647	*FLS*	F: TTAAAGGAAGGTCTCGGTGGCGAA
				R: TCATTGGTGACGATGAGTGCGAGT
11	NCBI	FJ969844	*Turnip Actin*	F: GCTCAGTCCAAGAGAGGTATTC
				R: GCTCGTTGTAGAAAGTGTGATG

Gene sequences retrieved from Brassica database (BRAD) and National Centre for Biotechnology (NCBI)

### Biological activity

#### Antioxidant activity

For the antioxidant studies, 1,1-diphenyl-2-picrylhydrazyl (DPPH) scavenging activity, reducing potential, phosphomolybdenum and chelating effects on ferrous ion methods were used following the procedure earlier reported (Thiruvengadam et al. 2014, 2016; Ghosh et al. 2015).

#### Antimicrobial activity

The pathogenic microorganisms of Gram-positive bacteria (*Staphylococcus aureus* and *Bacillus subtilis*), Gram-negative bacteria (*Pseudomonas aeruginosa* and *Escherichia coli*) and the fungi (*Candida albicans*, *Aspergillus niger* and *Fusarium oxysporum*) were used to evaluate antimicrobial activity. Methanolic extracts of transgenic and non-transgenic roots (100 mg mL^−1^) was tested for antimicrobial activity. Antimicrobial tests were carried out by the NCCLS disc diffusion method as previously described by Thiruvengadam et al. (2014). For the antimicrobial tests, paper discs (10 mm in diameter) were individually impregnated with 50 µL of 100 mg mL^−1^ plant extract and placed on the inoculated agar. For the positive control, paper discs were impregnated with 50 µL of chloramphenicol or thymol and plates were incubated at 37 °C for 18–24 and 24–48 h for bacterial and fungal plates, respectively. Relative percentage inhibition of the test extract = 100 × (*X* − *Y*)/(*Z* − *Y*), where *X* is the total area of inhibition of the test extract, *Y* is the total area of inhibition of the solvent, and *Z* is the total area of inhibition of the standard drug. The total area of the inhibition was calculated by using area = *πr* ^2^; where, *r* = radius of the zone of inhibition.

#### Cytotoxic screening of MTT assay

Two human cancer cell lines, namely colon HT-29 (human colorectal adenocarcinoma) and oestrogen-dependent breast MCF-7 (human breast adenocarcinoma) cancer cells were used for cytotoxicity screening of the hairy roots and non-transformed root extracts in turnip. The cell lines cultured in DMEM medium supplemented with 10 % FBS and incubated at 37 °C in a humidified incubator with 5 % CO_2_. The cells were washed with PBS and then treated with Trypsin. The culture was centrifuged, and the pellet was re-suspended in fresh medium. Briefly, cells (5 × 10^3^ cells well^−1^) were plated in 96 well plates and treated with different concentrations of transgenic and non-transgenic root extracts (12.5, 25, 50, 100 and 200 µg mL^−1^) for 48 h. The solvent DMSO treated cells served as control. Cell viability was determined using MTT [3-(4, 5-dimethylthiazol-2-yl)-2, 5-diphenyl tetrazolium bromide] colorimetric assay was performed to evaluate the cytotoxicity according to the method described by Gabr et al. (2016) with slight modifications. Cells were then treated with MTT reagent (20 µL well^−1^) for 4 h at 37 °C and then DMSO (200 µL) was added to each well to dissolve the formazan crystals. The optical density (OD) was recorded at 492 nm in a microplate reader. The percentage inhibition of cell growth was calculated using the following formula: 100 − (A_sample_/A_control_ × 100).

### Experimental design and data analysis

All experiments were performed in triplicate and each experiment was repeated three times. The data were expressed as mean ± standard deviation (SD). One-way ANOVA analysis followed by Duncan’s test was used to determine significant differences (*P* ≤ 0.05). All statistical analyses were performed using the SPSS Ver. 20 statistical software package (SPSS Inc., Chicago, USA).

## Results

### Establishment of transgenic hairy root cultures

Two hundred and fifty explants from in vitro seedlings of turnip, including explants of leaves, hypocotyls, and roots segments (750 explants in total), were inoculated with *A. rhizogenes* strain KCTC 2703. In all cases, 50 explants were used as controls. However, leaf explants more readily produced hairy roots in response to inoculation by *A. rhizogenes* (89 %) than did hypocotyl (11 %) explants (*P* ≤ 0.05). In contrast, root explants failed to respond within 11 days of inoculation. No hairy roots or calli formed from control explants. Initially, root protuberances developed within 10–12 days of inoculation at the wounded edges of leaf explants, with hairy roots developing within 21 days of inoculation. After 3 weeks of culture, the hairy roots that measured about 1.0–2.0 cm in length were excised and transferred to liquid medium for suspension culture. The established hairy roots exhibited typical morphological characteristics with rapid growth on phytohormone-free medium, lack of geotropism, and extensive lateral branching.

### Molecular analysis of *rolC* gene integration

**Fig. 1:**
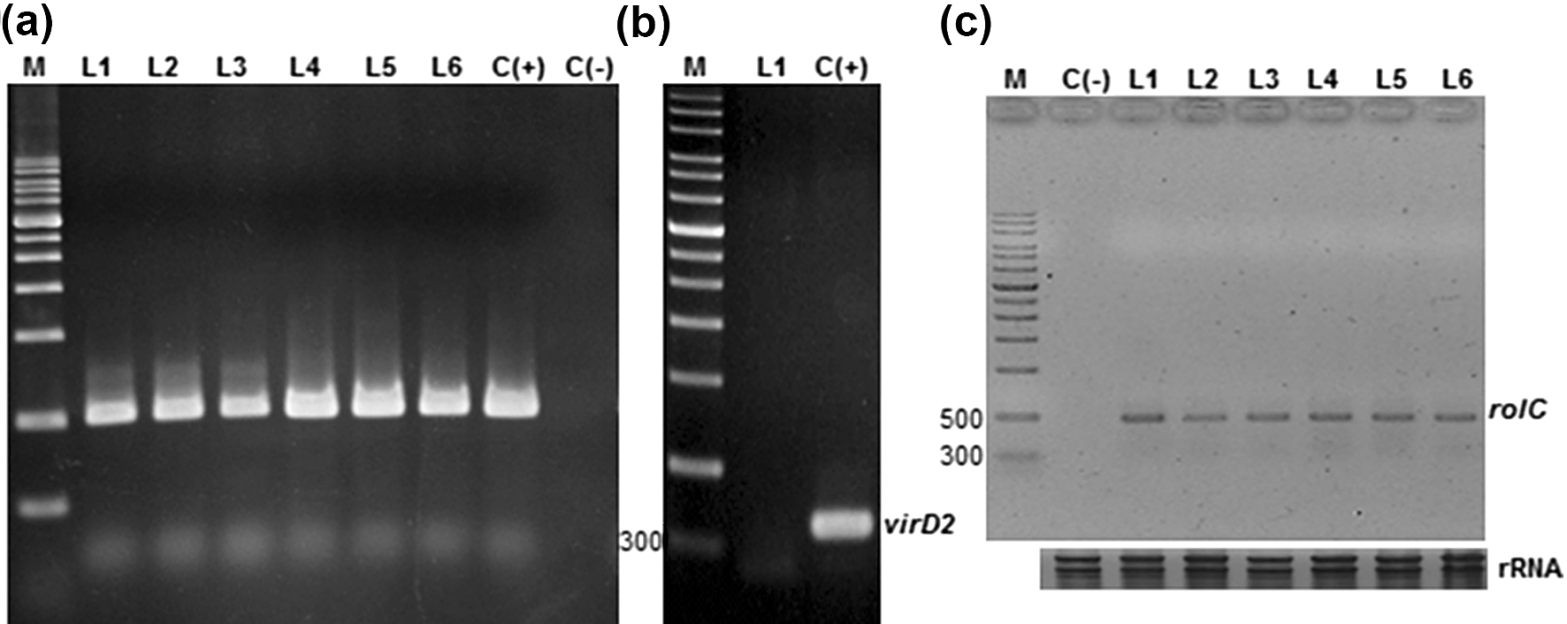
Molecular confirmation of hairy root cultures in turnip. **a** PCR analysis of the *rolC* gene in the hairy root lines of turnip [lane M DNA ladder marker, transgenic root lines induced by *A. rhizogenes* L1–L6, plasmid DNA from KCTC 2703 C(+), roots from a non-transformed control plant C(−)]. **b** PCR analysis of the *virD2* gene in the pRiKCTC2703 and hairy root lines of turnip [lane M DNA ladder marker, L1 transgenic root lines induced by *A. rhizogenes*, C(+) plasmid DNA]. **c** RT-PCR analysis of the *rolC* gene expression using *rolC* primers in the hairy root lines of turnip [lane M DNA ladder marker, L1–L6 transgenic root lines induced by *A. rhizogenes*, C(−) roots from a non-transformed plant]

To evaluate the molecular confirmation of the transgenic hairy roots, we used a PCR-based analysis that targeted the *A. rhizogenes rolC* and *virD2* genes. The *rolC* gene (located on the pRi TL-DNA segment) was used to confirm the presence of the fragment in the root plant genome, and the *virD2* gene (located outside the T-DNA of *A. rhizogenes*) was used to confirm the total absence of *Agrobacterium* in the hairy root cultures. Integration of *A. rhizogenes* Ri plasmid T-DNA into turnip genome was confirmed by PCR and RT-PCR. PCR and RT-PCR analysis using a *rolC*-specific primer with an amplicon of ~500 bp confirmed the transgenic roots. The fragment of *rolC* was observed in the amplified DNA of all six hairy root lines (lanes L1–L6, Fig. 1a, c), just as it was in a positive control (amplification product of plasmid DNA from KCTC 2703) (lane C(+), Fig. 1b). In contrast, no such amplification product was found in the DNA isolated from non-transformed control roots (lane C(−), Fig. 1a, c). No fragments of the *virD2* gene were found in the hairy root lines (lane L1, Fig. 1b). This result indicates that pRi T-DNA fragments of *A. rhizogenes* were successfully integrated into the genome of turnip without bacterial residues.

### Factors influencing the growth index and biomass accumulation of hairy root cultures

**Fig. 2:**
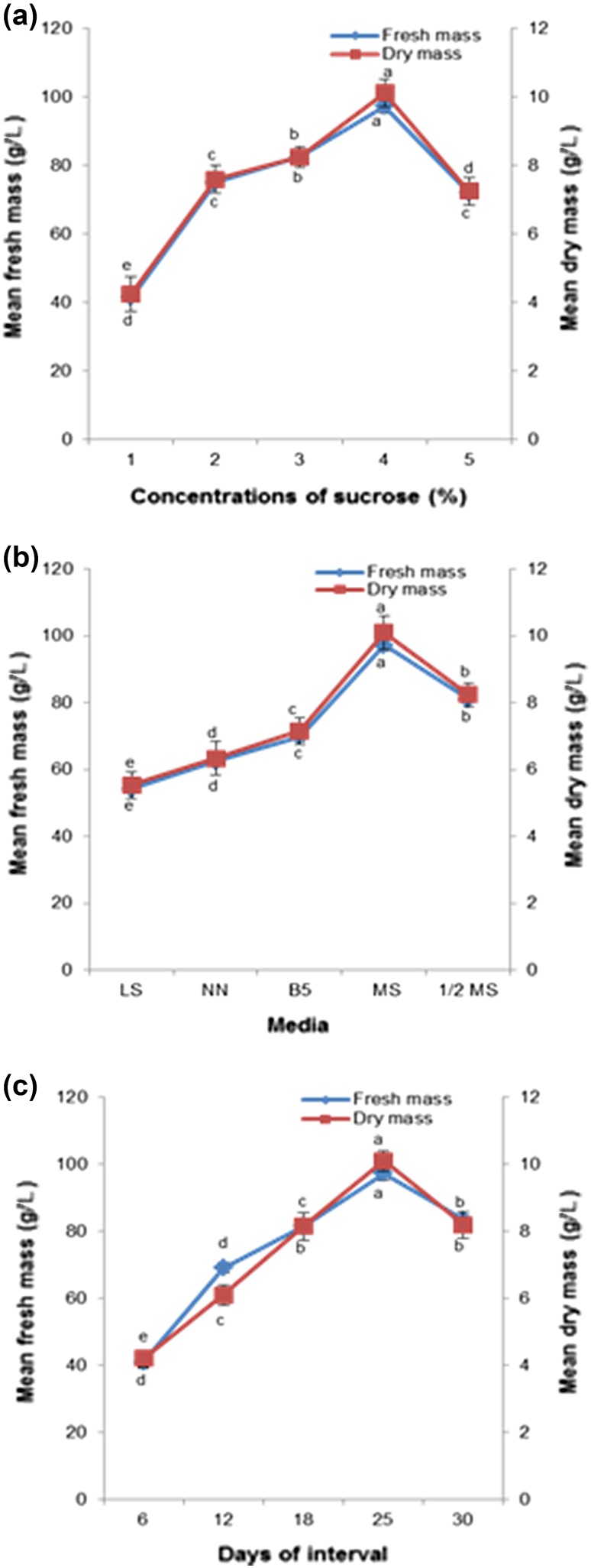
Factors influencing the biomass accumulation of hairy root cultures in turnip. **a** Biomass accumulation of hairy root cultures as affected by different concentrations of sucrose in the MS medium. **b** Biomass accumulation of hairy root cultures as affected by different media. **c** Time profile of hairy root cultures in MS medium supplemented with 4 % sucrose. Mean ± standard deviation of three replicates followed by the *same letters* are not significantly different according to Duncan’s multiple range test at *P* ≤ 0.05

Sucrose is the most important carbon source for plant tissue cultures, and it serves as the main energy source, and used for biomass accumulation and biosynthesis of secondary metabolites. We studied the effects of sucrose concentration (1–5 %) in MS medium on the growth of hairy roots (Fig. 2a). In the present investigation, MS medium supplemented with 4 % sucrose produced 97.25 g L^−1^ fresh mass and 10.11 g L^−1^ dry mass (*P* ≤ 0.05) accumulation. Hairy root growth considerably decreased in media containing concentrations of sucrose either above or below the 4 % level. In the present investigation, different media of full-strength and half-strength MS, B5, NN, and LS were employed in hairy root culture and the results show that MS medium was greater for biomass accumulation (*P* ≤ 0.05; Fig. 2b). The maximum accumulation of biomass was found in the full-strength MS medium, followed by half-strength MS medium (*P* ≤ 0.05). Figure 2c shows that the complete growth cycle could be separated into four phases: an adaptive phase (0–6 days), an exponential phase (6–24 days), a stationary phase (24–30 days), and a decline phase (after 30 days). The highest biomass accumulation appeared in the 24–26th day when the biomass reached a peak of 97.25 g L^−1^ FM and 10.11 g L^−1^ DM (*P* ≤ 0.05). A 9.2-fold increment was evident when compared with initial inoculum fresh biomass. A higher amount of biomass accumulation was noted in MS medium augmented with 4 % sucrose at 25 days of culture.

### Evaluation of GSLs profiles in hairy roots and non-transformed roots

**Fig. 3:**
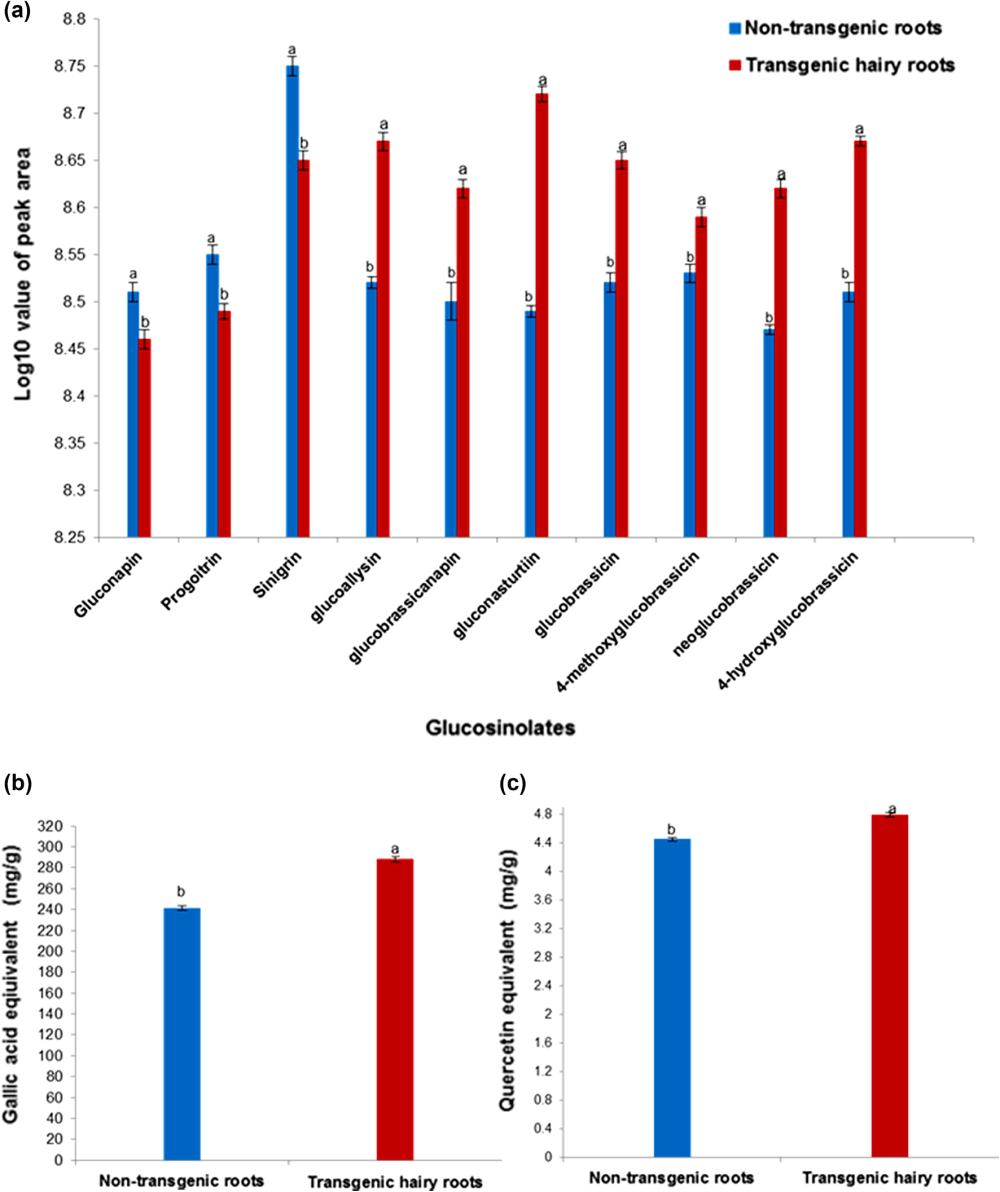
Evaluation of phytochemicals (GSLs and phenolic compounds) content of transgenic and non-transgenic roots in turnip. **a** GSLs identified by UHPLC-TQMS. **b** Determination of total phenolic content. **c** Determination of total flavonoid content. Mean ± standard deviation of three replicates followed by the *same letters* are not significantly different according to Duncan’s multiple range test at *P* ≤ 0.05

GSLs were identified by UHPLC-TQMS in hairy roots and non-transformed roots of turnip. Ten GSLs, including five AGSLs (gluconapin, progoitrin, sinigrin, glucoallysin, and glucobrassicanapin), aromatic GSLs (gluconasturtiin), and four IGSLs (glucobrassicin, 4-methoxyglucobrassicin, neoglucobrassicin, and 4-hydroxyglucobrassicin) were compared (Fig. 3a; Table 2). The amount of glucoallysin, glucobrassicanapin, gluconasturtiin, glucobrassicin, 4-methoxyglucobrassicin, neoglucobrassicin, and 4-hydroxyglucobrassicin significantly increased in hairy roots compared to non-transformed roots (*P* ≤ 0.05). Gluconapin, progoitrin, and sinigrin levels decreased in hairy roots compared to non-transformed roots (*P* ≤ 0.05).

**Table 2:** Glucosinolates identified in turnip

RT (min)	Trivial names	Semisystematic names of R-groups	Parental amino acids	Compound groups	*m*/*z*		Molecular mass^a^
					[M − H]^−^	[M + H]^+^	
2.597	Progoitrin	(2R)-2-Hydroxy-3-butenyl	Methionine	Aliphatic	309	311	310
3.306	Sinigrin	2-Propenyl	Methionine	Aliphatic	279	281	280
3.887	Gluconapin	3-Butenyl	Methionine	Aliphatic	293	295	294
4.306	Glucobrassicanapin	Pent-4-enyl	Methionine	Aliphatic	307	309	308
4.514	Glucoallysin	5-Methylsufinylpentyl	Methionine	Aliphatic	371	373	372
4.523	Glucobrassicin	3-Indolymethyl	Tryptophan	Indolyl	368	370	369
4.622	4-Hydroxyglucobrassicin	4-Hydroxy-3-indolylmethyl	Tryptophan	Indolyl	384	386	385
4.805	4-Methoxyglucobrassicin	4-Methoxy-3-indolylmethyl	Tryptophan	Indolyl	397	399	398
4.957	Gluconasturtiin	2-Phenethyl	Phenylalanine	Aromatic	343	345	344
5.251	Neoglucobrassicin	*N*-methoxy-3-indolylmethyl	Tryptophan	Indolyl	398	400	399

*RT* retention time

^a^Bennett et al. (2004)

### Evaluation of total phenolic and flavonoid content (TPC and TFC) in hairy roots and non-transformed roots

Total phenolic and flavonoid content were higher in hairy roots than that in non-transformed roots (*P* ≤ 0.05; Fig. 3b, c). The total phenolic content of hairy roots was 287.89 mg g^−1^, and their total flavonoid content was 4.79 mg g^−1^. The total phenolic content of non-transformed roots was 241.27 mg g^−1^, and their total flavonoid content was 4.45 mg g^−1^.

### Evaluation of individual phenolic compound profiles in hairy roots and non-transformed roots

The qualitative and quantitative analyses of phenolic compounds from turnip hairy roots and non-transgenic root extracts were performed using UPLC (Table 3). The phenolic compounds in turnip root extracts were identified by comparison of their retention time and UV spectra with that of authentic standards, and the quantitative data were calculated from calibration curves. Both non-transformed and hairy roots contained hydroxybenzoic acid, hydroxycinnamic acid and flavonols (Table 3). Hairy roots contained higher level of hydroxybenzoic acid (459.31 μg g^−1^), hydroxycinnamic acid (582.09 μg g^−1^) and flavonols (941.46 μg g^−1^) than non-transformed plants (*P* ≤ 0.05), which produced lower amounts of hydroxybenzoic acid (413.81 μg g^−1^), hydroxycinnamic acid (518.97 μg g^−1^) and flavonols (867.38 μg g^−1^). The content of *p*-Hydroxybenzoic, protocatechuic and syringic acids were significantly higher in hairy roots than that in non-transformed roots (*P* ≤ 0.05; Table 3). Ferulic and chlorogenic acids were higher in transgenic hairy roots than that in non-transformed roots (*P* ≤ 0.05; Table 3). Gentisic, *p*-coumaric, *m*-coumaric and *t*-cinnamic acids were higher in non-transformed roots than in hairy roots (*P* ≤ 0.05; Table 3). Catechin, myricetin, quercetin, rutin were significantly higher in hairy roots than that in non-transformed roots (*P* ≤ 0.05; Table 3). The contents of kaempferol, naringenin, biochanin A, hesperidin and formononetin were higher in non-transformed roots than in hairy roots (*P* ≤ 0.05; Table 3).

**Table 3:** Major phenolic constituents identified by UPLC analysis from transgenic hairy roots and non-transgenic roots of turnip

No.	Group of phenolic compounds	Concentration (µg g^−1^ DM)	
		Non-transgenic roots	Transgenic hairy roots
	Hydroxybenzoic acid		
1	*p*-Hydroxybenzoic acid	151.25 ± 1.02^b^	178.25 ± 1.04^a^
2	Protocatechuic acid	5.60 ± 0.33^b^	17.27 ± 0.35^a^
3	Syringic acid	75.38 ± 1.03^b^	89.17 ± 0.62^a^
4	Gentisic acid	181.58 ± 1.19^a^	174.62 ± 1.10^b^
	Total	413.81^b^	459.31^a^
	Hydroxycinnamic acid		
5	*p*-Coumaric acid	125.25 ± 1.04^a^	94.50 ± 1.29^b^
6	Ferulic acid	265.00 ± 2.16^b^	326.37 ± 1.10^a^
7	Chlorogenic acid	84.12 ± 0.85^b^	135.75 ± 1.04^a^
8	*m*-Coumaric acid	17.95 ± 0.85^a^	10.17 ± 0.62^b^
9	*t*-Cinnamic acid	26.65 ± 0.88^a^	15.30 ± 0.53^b^
	Total	518.97^b^	582.09^a^
	Flavonols		
10	Myricetin	80.17 ± 0.85^b^	115.25 ± 0.95^a^
11	Quercetin	95.75 ± 1.19^b^	110.17 ± 0.92^a^
12	Kaempferol	89.32 ± 1.13^a^	65.87 ± 0.85^b^
13	Catechin	425.00 ± 1.12^b^	451.50 ± 1.08^a^
14	Naringenin	19.50 ± 0.91^a^	12.40 ± 0.63^b^
15	Rutin	109.50 ± 0.70^b^	152.25 ± 1.04^a^
16	Biochanin A	12.77 ± 0.75^a^	7.22 ± 0.69^b^
17	Formononetin	3.67 ± 0.23^a^	2.53 ± 0.13^b^
18	Hesperidin	31.70 ± 0.62^a^	24.27 ± 0.60^b^
	Total	867.38^b^	941.46^a^

Mean ± standard deviation within a row followed by the same letters are not significantly different according to Duncan’s multiple range test at *P* ≤ 0.05

*nd* not determined

### Evaluation of phytochemicals (GSLs and phenolic compounds) biosynthetic gene expression in hairy roots and non-transformed roots

**Fig. 4:**
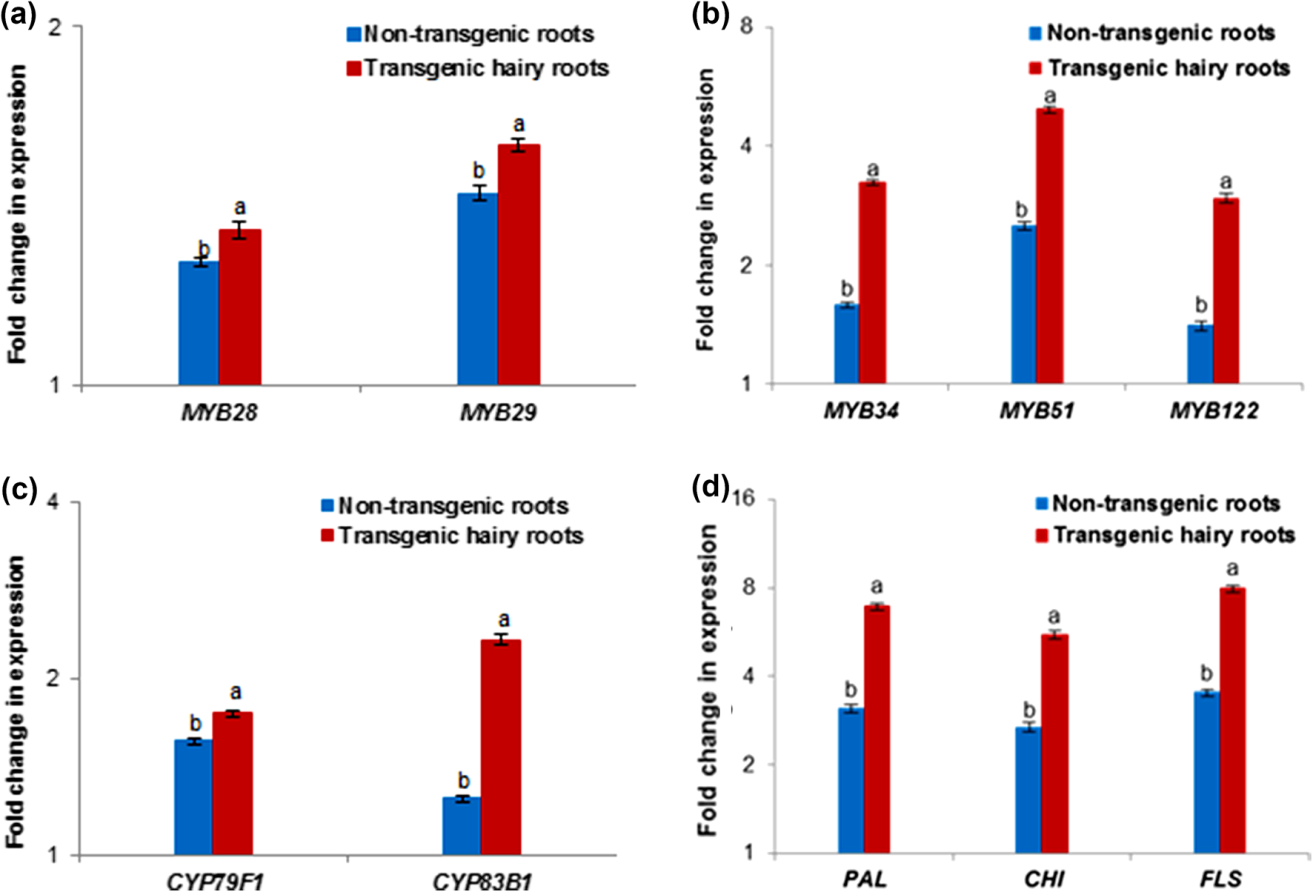
The expression level of genes associated with GSLs and phenolic compound biosynthesis in transgenic and non-transgenic roots of turnip. **a** *MYB28* and *MYB29*. **b** *MYB34*, *MYB51* and *MYB122*. **c** *CYP83B1* and *CYP79F1*. **d** *PAL*, *CHI* and *FLS* gene expression. All values showed in the histograms represented as log2 fold change in mRNA levels compared to the control, which assigned as 1. Mean ± standard deviation of three replicates followed by the *same letters* are not significantly different according to Duncan’s multiple range test at *P* ≤ 0.05

Gene expression investigations provide further insight into how GSLs biosynthesis is regulated by metabolic pathways in transgenic hairy roots and non-transgenic roots. We determined the relative transcript levels of the essential genes involved in the biosynthesis of GSLs in transgenic hairy roots and non-transgenic roots of turnip. The expression levels of genes related to GSL biosynthesis (*BrMYB28*, *BrMYB29*, *BrMYB34*, *BrMYB51*, *BrMYB122*, *CYP79F1*, and *CYP83B1*) were higher in transgenic hairy roots than that in non-transgenic roots (*P* ≤ 0.05; Fig. 4a, b, c). Similarly, the individual GSLs (glucoallysin, glucobrassicanapin, gluconasturtiin, glucobrassicin, 4-methoxyglucobrassicin, neoglucobrassicin and 4-hydroxyglucobrassicin) contents increased in transgenic hairy roots compared to that in non-transgenic roots. The expression levels of phenolic biosynthetic genes (*PAL*, *CHI* and *FLS*) were higher in transgenic roots than non-transgenic roots (*P* ≤ 0.05; Fig. 4d). Consistently, the phenolic and flavonoid contents were increased in transgenic hairy roots compared to that in non-transgenic roots. This is the first report to compare the phytochemicals (GSLs and phenolic) content and corresponding gene transcriptional profiles (*BrMYB28*, *BrMYB29*, *BrMYB34*, *BrMYB51*, *BrMYB122*, *CYP79F1*, *CYP83B1, PAL*, *CHI* and *FLS*) of transgenic hairy roots and non-transgenic roots in turnip.

### Evaluation of biological activity in hairy roots and non-transformed roots

#### Antioxidant activity

**Fig. 5:**
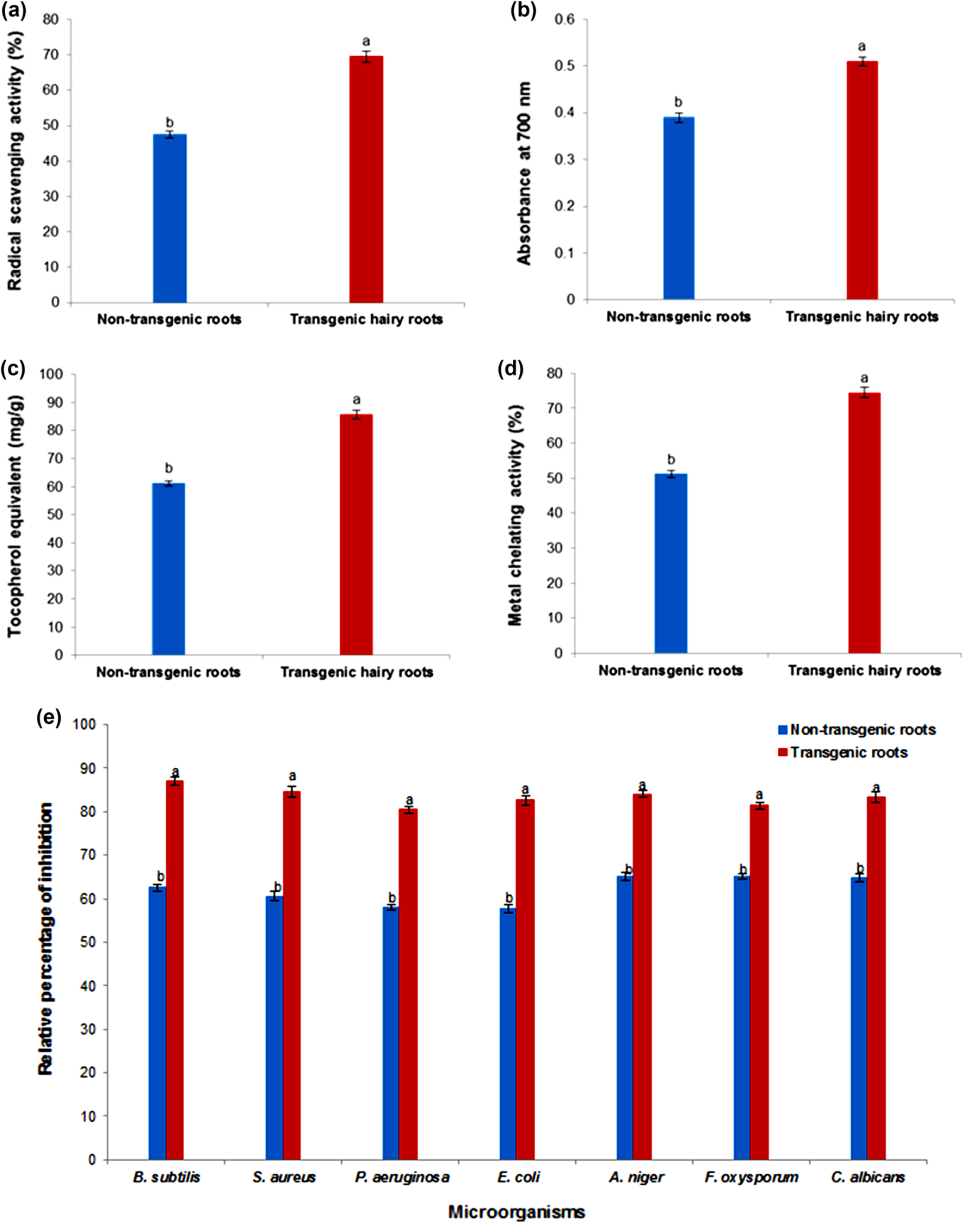
Evaluation of antioxidant and antimicrobial activity of transgenic and non-transgenic roots in turnip. **a** Free radical-scavenging activity by the DPPH method. **b** Reducing power. **c** Phosphomolybdenum method. **d** Metal ion chelating activity. **e** Antimicrobial activity. Mean ± standard deviation of three replicates followed by the *same letters* are not significantly different according to Duncan’s multiple range test at *P* ≤ 0.05

The antioxidant potential of hairy roots and non-transformed roots was determined using free radical scavenging, reducing potential, and phosphomolybdenum assays. To evaluate the free radical scavenging ability of the hairy roots and non-transformed roots extracts, we performed DPPH assays (Fig. 5a) to quantify the capacity of an electron transfer and hydrogen-atom abstraction reaction, which is a marginal reaction pathway. The antioxidant activity exhibited by hairy roots was higher (69.50 %) than that shown by non-transformed roots (47.50 %; *P* ≤ 0.05) (Fig. 5a). The reducing potential results for turnip extracts suggested that hairy roots had a greater antioxidant potential than non-transformed roots (*P* ≤ 0.05; Fig. 5b). The antioxidant capacity of the hairy roots and non-transformed root extracts was determined using the phosphomolybdenum method, which is based on the reduction of Mo (VI) to Mo (V) by the sample analyte, and the subsequent formation of green phosphate/Mo (V) compounds with a maximum absorption at 695 nm. The antioxidant capacity of the hairy root extracts was 85.61 mg g^−1^ and that of the non-transformed root extracts was 61.15 mg g^−1^ (Fig. 5c). The chelating agent may inhibit radical generation by stabilizing transition metals, thus reducing free radical damage. Figure 5d shows the percentage of metal scavenging capacity was exhibited higher in hairy roots (74.50 %), compared with non-transformed roots (51.10 %). In the present investigation, hairy roots showed higher antioxidant activity than the non-transformed roots (*P* ≤ 0.05).

#### Antimicrobial activity

The hairy roots and non-transgenic roots of turnip exhibited varying antimicrobial (antibacterial and antifungal) activity, as shown by the growth IZs (Fig. 5e). The results from the disc diffusion method indicated that both hairy roots and non-transformed root extracts had comparable antibacterial effects against Gram-positive and Gram-negative bacteria. Hairy roots exhibited higher activity against both Gram-positive and Gram-negative bacteria than non-transformed roots (*P* ≤ 0.05). Gram-positive bacteria (*S. aureus* and *B. subtilis*) were markedly inhibited compared to Gram-negative bacteria (*P. aeruginosa* and *E. coli*) (*P* ≤ 0.05). Figure 5e depicts the results from the disc diffusion method against the fungal strains. Hairy roots showed better antifungal activity than non-transformed roots. These results were compared with the positive control (chloramphenicol and thymol) for evaluating their relative percentage of inhibition (Fig. 5e).

#### Anticancer activity

**Fig. 6:**
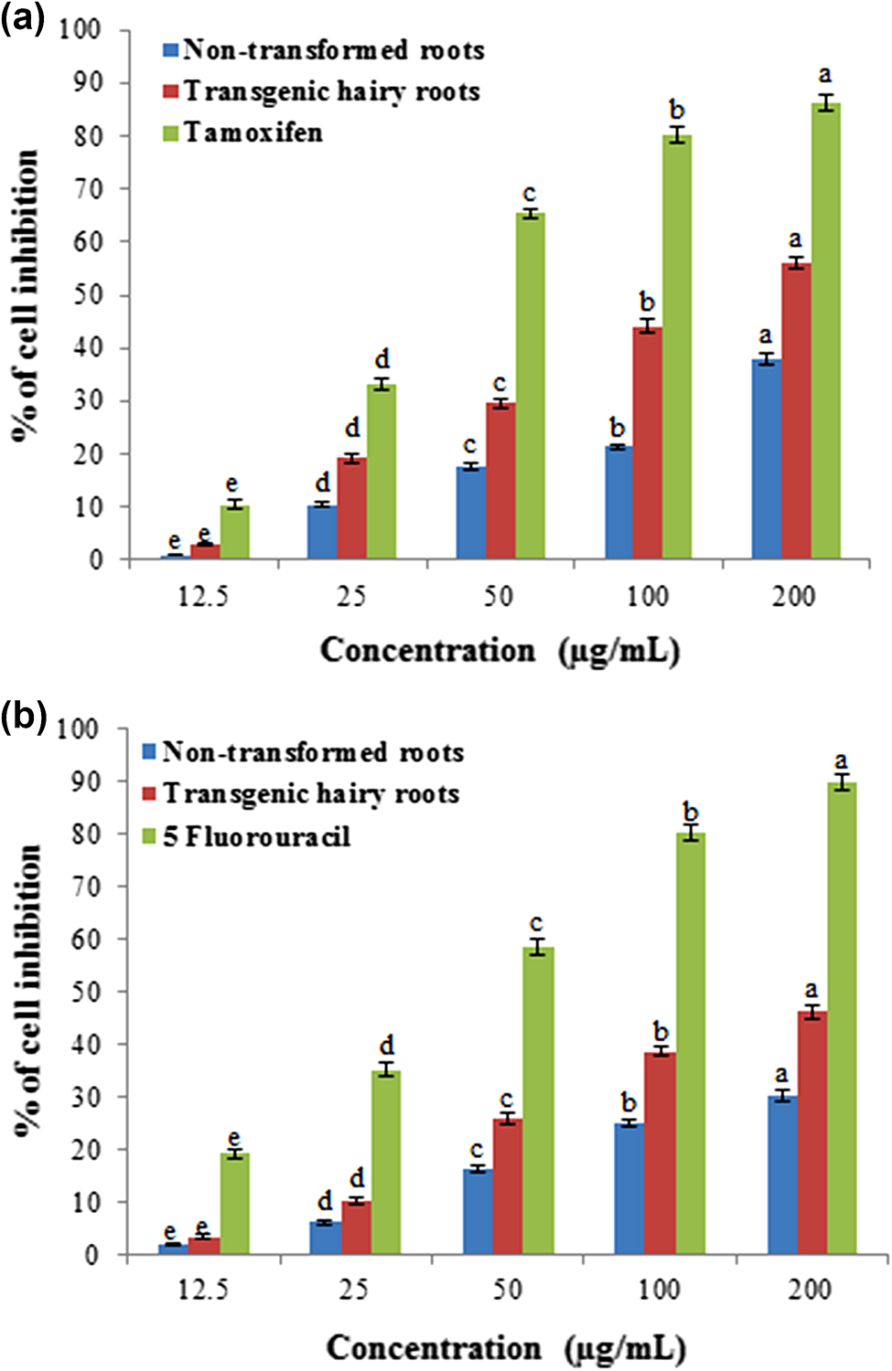
Percent cell inhibition of transgenic and non-transgenic roots in turnip extracts on MCF-7 and HT-29 cell lines. **a** MCF-7. **b** HT-29. Mean ± standard deviation of three replicates followed by the *same letters* are not significantly different according to Duncan’s multiple range test at *P* ≤ 0.05

Screening of the anticancer activities against MCF-7 and HT-29 cell lines in hairy roots and non-transgenic roots of turnip. The inhibitory properties of these extracts are compared with standard tamoxifen for MCF-7 cell line and 5-fluorouracil for HT-29 cell line (Fig. 6a, b), respectively. The percentage cancer cell inhibition profiles were found to be concentration dependent and the greater inhibition was higher concentration (200 µg mL^−1^). MCF-7 cell lines grown in DMEM, when subjected to different concentrations of hairy roots extracts resulted in 55.98 % inhibition of MCF-7 cell death whereas non-transgenic roots displayed weak inhibition of 38.02 %. On the other hand, comparison with tamoxifen showed that 86.16 % MCF-7 cell line inhibition at the same tested dose (200 µg mL^−1^). However, at 50 µg mL^−1^ tested dose, 65.4 % inhibition was observed with tamoxifen, whereas only hairy root extracts has crossed 50 % inhibition, however, at 200 µg mL^−1^ tested dose (Fig. 6a). Hairy roots and non-transformed root extracts tested against HT-29 cell line resulted in anticancer activity profile than the standard 5-fluorouracil (Fig. 6b). A maximum concentration of 46.09 % inhibition was observed with hairy root extracts at a tested dose of 200 µg mL^−1^. MCF-7 cell line was greater inhibition than HT-29 cell line (Fig. 6a, b).

## Discussion

In this study, we found that leaf explants from in vitro seedlings of turnip were more successfully transformed by *A. rhizogenes* than hypocotyl or root explants. Similarly, leaf explants had a higher frequency of hairy root induction in *Momordica charantia* (Thiruvengadam et al. 2014), and *Astragalus membranaceus* (Jiao et al. 2015). PCR analyses of reference genes showed that most of the established hairy root lines carried the *rolC* gene in their genome but not the *virD2* gene, confirming the genetic transformation of the roots and the elimination of the hairy root-inducing bacteria. In the present study, MS medium supplemented with 4 % sucrose had high biomass accumulation. Our results are consistent with those of previous studies that found 4 % sucrose produced more biomass accumulation in hairy root cultures of *Stevia rebaudiana* (Fu et al. 2015). It has been reported that MS medium is favored biomass accumulation in hairy roots of *G. sylvestre* (Nagella et al. 2013), *M. charantia* (Thiruvengadam et al. 2014), and *Astragalus membranaceus* (Jiao et al. 2015). Previous studies have described hairy root induction and variations in growth level during different growth stages in *Nasturtium officinale* (Park et al. 2011), *G. sylvestre* (Nagella et al. 2013) and *M. charantia* (Thiruvengadam et al. 2014). Nagella et al. (2013) reported that the FM (0.5 g) of *G. sylvestre* hairy roots increased ~9.4-fold after 25 days of culture in hormone-free MS liquid medium. Our results suggest that hairy root cultures of turnip are promising for large-scale biomass production in liquid cultures.

Previously, it has been demonstrated that higher GSLs concentrations were observed in roots than in shoots at the same developmental stages of radish and cabbage (Bhandari et al. 2015). Our results reveal the GSLs content as well as the gene expression profiles of corresponding biosynthetic pathways in hairy roots and non-transgenic roots of turnip. We found that aromatic GSLs and IGSLs contents were prominent in hairy roots compared with that in non-transgenic roots. Consistent with our reports, gluconasturtiin, glucobrassicin, neoglucobrassicin, 4-hydroxyglucobrassicin, and 4-methoxyglucobrassicin were previously found to be leading in hairy roots of watercress (Park et al. 2011) and broccoli (Kim et al. 2013). Aromatic glucosinolate of glucotropaeolin was higher in hairy roots compared to callus, cell suspension culture and two-month-old plant leaves of *Tropaeolum majus* (Wielanek and Urbanek 1999). In our investigation, AGSLs of gluconapin, progoitrin, and sinigrin significantly decreased in hairy roots compared with that in non-transformed roots. Kastell et al. (2013a) reported that AGSLs content was considerably lower in hairy root cultures compared to roots of whole plants in *Arabidopsis*. In our study, the transcription factors associated with AGSLs (*MYB28, MYB29* and *CYP79F1*) was slightly high expression whereas IGSLs (*MYB34*, *MYB51*, *MYB122* and *CYP83B1*) biosynthesis was a significantly high expression in hairy roots compared with non-transgenic roots. Similar with our study, Kastell et al. (2013b) reported that IGSLs significantly increased in hairy root cultures of *B. rapa* and *S. alba*. Transcript levels of aliphatic biosynthetic genes (*MAM1*, *CYP79F1*, *CYP83A1*, *UGT74C1*, and *SUR1*) were significantly lower in hairy root cultures compared to roots of intact plants of *A. thaliana* (Kastell et al. 2013a). Over-expression of *CYP79F1* and *CYP79F2* in hairy roots was upregulated the transcript levels and also highly increased the content of short-chain AGSLs than control hairy root line of *A. thaliana* (Kastell et al. 2015).

The present investigation, phenolic compounds were higher in hairy roots than non-transformed roots. Consistently, Weremczuk-Jezyna et al. (2013) and Thiruvengadam et al. (2014) demonstrated that total phenolic and flavonoid content was higher in hairy roots than that in non-transformed roots of *Dracocephalum moldavica* and *M. charantia*. Previously it has been demonstrated that phenolic compound levels are 20-fold higher in extracts from hairy root cultures than that in the control normal plant of *Beta vulgaris* (Georgiev et al. 2010). Park et al. (2011) have demonstrated that both transformed and non-transformed roots of *Platycodon grandiflorum* had similar concentrations of phenolic compounds. Kim et al. (2009) stated that hairy roots produced higher amounts of catechin, rutin, quercetin, caffeic, chlorogenic, ferulic and gallic acids than non-transgenic roots of *Fagopyrum tataricum*. Similar to our reports, chlorogenic, protocatechuic, and ferulic acid content was higher in hairy roots than that in non-transformed roots of tomato (Singh et al. 2014). Consistently, hairy root cultures have been used for the large-scale production of chlorogenic acid derivatives in *Stevia rebaudiana* (Fu et al. 2015). The stimulation of phenolic and flavonoid biosynthetic genes are corresponds to the higher accumulation of phenolic and flavonoid contents in hairy roots. In our investigations, the transcription factors associated with phenolic and flavonoid (*PAL*, *CHI* and *FLS*) biosynthesis were highly expressed in hairy roots compared with non-transgenic roots. Similarly, the expression levels of phenolic and flavonoid (*PAL*, *CHI* and *FLS*) biosynthesis and also phenolic contents were highly expressed in hairy roots of *F. tataricum* (Thwe et al. 2013, 2014).

Antioxidant, antimicrobial and anticancer activity may be correlated with phenolic compounds (Lee et al. 2004; Daglia 2011). In our study, hairy roots showed higher antioxidant, antimicrobial and anticancer activity than non-transformed roots. Similarly, hairy roots of *Beta vulgaris* (Georgiev et al. 2010), *D. moldavica* (Weremczuk-Jezyna et al. 2013) and *M. charantia* (Thiruvengadam et al. 2014) exhibited high antioxidant activity. It has been reported that catechin, kaempferol, myricetin, naringin, quercetin, and rutin have good antimicrobial activity against human pathogenic microorganisms (Cushnie and Lamb 2005). Previously, it has been reported GSLs was significant anticancer activity and also antimicrobial activity, which might be beneficial in controlling human pathogens through the diet (Sisti et al. 2003; Hong and Kim 2008; Aires et al. 2009). Based on the present study, the levels of GSLs, phenolic compounds increased in transgenic hairy roots, which directly influenced their antioxidant, antibacterial, antifungal, and anticancer potential. Similar to our study, antimicrobial spectra of hairy root extracts were more effective against Gram-positive bacteria than Gram-negative bacteria (Wang et al. 2012). Previously, several reports corroborated with our study that hairy roots exhibited higher antibacterial and antifungal activity compared to non-transformed roots (Wang et al. 2012; Thiruvengadam et al. 2014). Syklowska-Baranek et al. (2012) reported that hairy roots exhibited higher anticancer activity compared to non-transformed roots of *Lithospermum canescens*. In this study shows the MTT assay resulted that hairy root cultures of turnip can inhibit the growth of breast and colon cancer cell lines. This inhibition of cell growth could be attributed to its GSLs and phenolic compounds content. Our results suggest that transformed hairy roots can be effectively used for the antioxidant, antimicrobial and anticancer treatments.

## Conclusions

Hairy root culture can be a valuable alternate approach for the large-scale production of health-benefitting secondary metabolites, especially GSLs and phenolic compounds in turnip. Hairy roots of turnip grew faster than control roots in standardized liquid culture conditions. Hairy roots produced a higher amount of GSLs, phenolic compounds and also exhibited gene expression levels higher when compared to non-transformed roots. The antioxidant, antimicrobial and anticancer activities were higher in hairy roots than that in non-transformed roots. The higher amounts of GSLs and phenolic compounds probably contribute to the marked antioxidant, antibacterial, antifungal and anticancer activity of hairy roots in turnip. Moreover, the results of the present investigation suggest the use of several biotic or abiotic elicitors to increase the content of GSLs and phenolic compounds in turnip hairy roots.

## Abbreviations

AGSLs: Aliphatic glucosinolates
DM: Dry mass
DPPH: 1,1-Diphenyl-2-picrylhydrazyl
FM: Fresh mass
GSLs: Glucosinolates
IGSLs: Indole glucosinolates
MTT: 3-(4, 5-Dimethylthiazol-2-yl)-2,5-diphenyl tetrazolium bromide
PCR: Polymerase chain reaction
qRT-PCR: Quantitative real-time polymerase chain reaction
RT-PCR: Reverse transcription polymerase chain reaction
TPC: Total phenolic contents
TFC: Total flavonoid contents
UHPLC-TQMS: Ultra-high-pressure liquid chromatography–triple quadrupole mass spectrometry
UPLC: Ultra-performance liquid chromatography
